# Structural Insights into the Regulation of a Key Tumor Suppressor

**DOI:** 10.1371/journal.pbio.0040040

**Published:** 2006-01-17

**Authors:** Liza Gross

The most common mutation in many human cancers disables p53, a key cell-growth regulator and tumor-suppressor protein. When a cell sustains DNA damage or some other stress, p53 activates genes involved in programmed cell death, cell cycle arrest, DNA repair, or other stress-induced responses. In healthy cells, p53 keeps a low profile, its numbers minimized by MDM2, an enzyme that marks p53 for rapid degradation with a ubiquitin tag through a process called ubiquitylation (also known as ubiquitination). As it happens, p53 also engineers its own destruction by including MDM2 in its list of transcriptional targets. How does the cell counteract this negative feedback loop and rescue p53 during times of stress?

Recent studies identified a deubiquitylating enzyme called HAUSP (herpesvirus-associated ubiquitin-specific protease) that can bind to p53, stabilize the protein, and promote cell death and cell growth arrest. But HAUSP can also deubiquitylate and stabilize MDM2. How can it stabilize both p53 and p53's nemesis? In a new study, Min Hu, Yigong Shi, and their colleagues used structural and mutational approaches to explore this paradox, and discovered that both p53 and MDM2 bind to the same location on the HAUSP protein domain in a mutually exclusive manner. Analysis of the molecular basis of their differential binding revealed that MDM2 binds HAUSP with a much higher affinity, and suggests how HAUSP may regulate the critically important p53–MDM2 pathway. [Fig pbio-0040040-g001]

**Figure pbio-0040040-g001:**
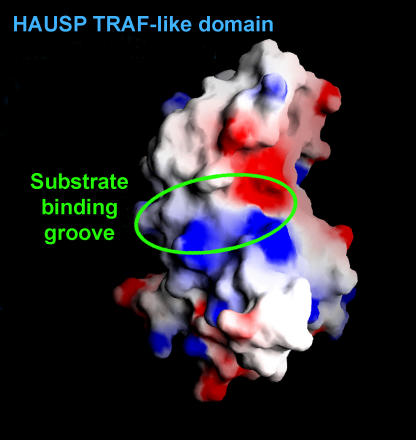
HAUSP TRAF-like domain contains a shallow surface groove, which corresponds to the region where the receptor peptides bind

Having determined that HAUSP recognizes p53 through a region of N-terminal residues within the TRAF-like domain in a previous study, the next step was determining the domain's structure. Proteins consist of linear sequences of amino acid residues joined end-to-end through peptide bonds. A polypeptide chain starts with an N-terminus (the domain at this end is called the N-terminal domain), and terminates at the C-terminus (so designated for its chemical properties). Hu et al. discovered that the HAUSP TRAF-like domain (named after a common domain in the TRAF family of cell signaling proteins) contains a shallow groove like the TRAF domain but lacks many of the same amino acids.

To study the structural details of HAUSP–p53 interactions, the authors mutated different amino acid residues in the p53 binding site and identified a short stretch of five amino acids required for binding. As efforts to obtain crystals of the p53–HAUSP complex were not successful, they generated protein chimeras—made of half p53 peptides and half HAUSP TRAF-like domain—to determine the complex's structure and mechanism of binding. This structure shows that the p53 peptide binds to HAUSP's shallow groove and reveals how many of the amino acids previously identified as important for HAUSP–p53 binding interact with specific p53 residues.

Hu et al. followed the same approach to reveal the mechanism of HAUSP–MDM2 interactions, and identified a ten–amino acid fragment in MDM2 as key to HAUSP recognition. Though this fragment bore little resemblance to the p53 peptide, HAUSP recognizes MDM2 with the same mechanism it uses to recognize p53—the N-terminus TRAF-like domain in the same shallow groove. As both p53 and MDM2 bind to the same groove on HAUSP, the authors realized their binding must be mutually exclusive and so staged a binding competition. And because MDM2 consistently formed stable complexes with HAUSP despite the presence of ten times more p53 peptides, it was clear that MDM2 binds to the deubiquitylating enzyme with a higher affinity.

Next, Hu et al. superimposed the two bound complexes. Though both peptides bind to the same shallow groove in the TRAF-like domain with the same orientation, MDM2's configuration allows more extensive interactions with HAUSP, which could explain its competitive binding edge. Interestingly, HAUSP's TRAF-like domain lacks most of the peptide-binding residues found in other TRAF proteins, suggesting that the HAUSP domain provides a new binding motif in the TRAF family. Though the HAUSP binding sites of p53 and MDM2 do not have obvious sequence similarity, the authors did find a common four-residue motif within them that is also present in a different HAUSP-binding protein (EBNA1). With knowledge of this motif, researchers can scan sequence databases for additional candidate HAUSP targets.

But how does recognition and binding mediate deubiquitylation? After solving the structure of a larger HAUSP fragment (including the catalytic domain, responsible for deubiquitylation, and the peptide-binding domain), the authors constructed a model of how HAUSP might recognize a ubiquitylated MDM2 protein. A small peptide fragment of MDM2 stabilizes binding to the TRAF-like domain while a separate HAUSP domain binds ubiquitin, and then cleaves the ubiquitin tag, promoting deubiquitylation.

These results suggest that HAUSP likely targets MDM2 under normal physiological conditions, and provide a valuable framework for probing the function of the p53–MDM2 pathway. The differential binding properties of p53 and MDM2 also suggest promising drug-screening targets. Given MDM2's negative impact on p53, it may be that inhibiting HAUSP, and thus MDM2, could counteract mutations that interfere with p53 function, and give this tumor suppressor the boost it needs to do its job.

